# Agreement between subjective gait assessment and markerless video gait‐analysis in endurance horses

**DOI:** 10.1111/evj.14516

**Published:** 2025-04-21

**Authors:** Mariaelena de Chiara, Chiara Montano, Andrea De Matteis, Livia Guidi, Francesco Buono, Luigi Auletta, Chiara Del Prete, Maria Pia Pasolini

**Affiliations:** ^1^ Department of Veterinary Medicine and Animal Production University of Naples Federico II Naples Italy; ^2^ Freelance Veterinary Practitioner Avellino Italy; ^3^ Department of Veterinary Medicine and Animal Science (DIVAS) University of Milan Lodi Italy

**Keywords:** endurance, equine lameness, gait asymmetry, horse, markerless video gait analysis

## Abstract

**Background:**

Subjective evaluation of gait by official endurance veterinarians (OEVs) is used to determine ‘fitness‐to‐compete’ in horses participating in endurance competitions. Objective gait analysis systems could aid in quick and verifiable judgements.

**Objectives:**

To assess the agreement between objective analysis of head and pelvis vertical movement asymmetry performed with a markerless artificial intelligence motion tracking system (AI‐MTS) and subjective lameness assessment performed by an accredited FEI OEV to judge horse gaits.

**Study Design:**

Cross‐sectional.

**Methods:**

During three endurance competitions, 110 horses were enrolled. The OEV performed 188 gait examinations, which were simultaneously recorded with a smartphone. The vertical motion asymmetry of the head and pelvis was later analysed from the videos through the AI‐MTS application. The gaits were scored as ‘no asymmetry’, ‘mild asymmetry’ or ‘severe asymmetry’. The agreement was evaluated using Fleiss' multi‐rater kappa statistic (*κ*).

**Results:**

The overall agreement between the two methods was fair (*k* = 0.26, *p* < 0.001). Within the three gait asymmetry categories, substantial agreement was obtained for the ‘severe’ (*k* = 0.75, *p* < 0.001) category, fair agreement was detected for the ‘no asymmetry’ category (*k* = 0.25, *p* < 0.001), and no agreement was identified for the ‘mild’ category (*k* = 0.13, *p* = 0.08).

**Main Limitations:**

Comparison between AI‐MTS and a single OEV; absence of a tripod during video recording; and video recording from a different point of view than the OEVs.

**Conclusions:**

Mild asymmetry was the most challenging gait category to identify. Substantial agreement between the subjective lameness evaluation by OEV and AI*‐*MTS assessment was observed for the ‘severe’ category. AI‐MTS may be a helpful tool to assist OEVs in decision‐making during endurance competitions.

## 1 INTRODUCTION

During endurance competitions, horses compete over distances ranging from 30 to 160 km in 24 h.^1^ In the vetting area, one or more official endurance veterinarians (OEVs) must establish whether they are ‘fit‐to‐compete’ to continue the races, applying uniform standards to assess the horse's fitness promptly.^1^, ^2^ Lameness is the most common reason for exclusion from endurance competitions.^3^, ^4^, ^5^ However, previous studies reported a low reliability of veterinary subjective evaluations of lameness in horses.^6^, ^7^, ^8^

Markerless smartphone applications could be particularly suitable in clinical practice, an example of which is the artificial intelligence (AI) markerless motion tracking system (AI‐MTS) (Sleip).^9^, ^10^, ^11^ The AI behind the AI‐MTS, Computer Vision, recognises the symmetry of specific points of a moving object on simple video footage without using any marker or sensor on the horse.^10^ This application has been found to have similar performance as an optical motion capture multicamera system using skin‐attached reflective markers.^12^ Specifically, the AI‐MTS identifies vertical motion asymmetry of the head and pelvis during the stride,^13^ and it allows for the analysis of trotting horses both during straight‐line locomotion and circling movements on various surfaces.^12^ The AI‐MTS allows video recordings made with a smartphone to be re‐examined in slow‐motion mode, with zooming on the horse and simultaneous observation of the vertical motion waves of the head and pelvis. Finally, it requires internet access for video processing and the optional use of a tripod during recording. Testing of this AI‐MTS in endurance horses has not yet been published.

The aim of this study was to evaluate the agreement between the objective analysis performed with the AI‐MTS and the visual lameness assessment carried out by an OEV. We anticipated that video recording would not interfere with the OEV examination and hypothesised that the overall agreement between the AI‐MTS and the OEV's lameness evaluation would be moderate to substantial in cases of more pronounced asymmetry and slight to fair in cases of mild gait asymmetry.

## 2 MATERIALS AND METHODS

### 2.1 Animals

In this study, a convenience sample of 110 horses was analysed. All horses included were of the purebred Arabian and Anglo‐Arabian breeds, with a median age of 6 years (4–18 years). The study was carried out during three Italian regional endurance competitions, including 30 (debutantes), 60 (CEN A) and 90 (CEN B) km categories, held between April and August 2023.

### 2.2 Data collection

Horses were prospectively recruited during physical examinations at the vetting area, and their gait was recorded and analysed through the AI‐MTS (v1.3.1, Sleip) using a smartphone (iPhone 13 Pro, iOs 16, Apple Inc.). The video streams were first recorded via the AI‐MTS, saved locally, and later uploaded to the software for data processing. Gait examinations were performed at the end of each loop.^14^ For the category 30 (debutantes) km, all videos were recorded only at the end of the competition; for the category 60 km (CEN A), videos were recorded both after one loop and at the end of the competition; for the category 90 km (CEN B), videos were recorded both at the end of the two loops and at the end of the competition. Whenever the OEV questioned a horse's fitness to continue after it had trotted once and a first score had been assigned, the horse was immediately re‐trotted to allow a better assessment, according to FEI regulations, and the operator recorded an additional video.^14^

The gait analysis protocol was the same for all the enrolled horses. Each horse was transferred to the vetting area without the saddle, and its heart rate recovery, metabolic status, gait, and general body condition were evaluated.^1^, ^14^ For gait examination, horses were trotted by a staff member on a loose lead, 40 m out and 40 m back for a total of 80 m. The videos were recorded with the horses always trotting on a hard, level surface arranged at the veterinary gate. The OEV assigned the horse an asymmetry gait score specified by the FEI regulations, that is, A for soundness, B for mild lameness and C for severe lameness. For criterion C, horses were defined as those exhibiting a consistently irregular gait observable at both the walk and trot, likely causing discomfort to the horse or compromising its future athletic performance.^1^, ^14^ Horses were excluded from the competition if veterinarians reported a type C gait.^1^, ^14^ One of the research team members performed all of the visual lameness evaluations (L.G., FEI‐certified OEV, with over 10 years of experience). The OEV evaluated the horses' gait and reported the subjective assessment in a written form. The OEV evaluation took into consideration the gait as a whole, without specifying which limb or how many limbs were affected. In cases of multi‐limb asymmetry, the OEV reported the higher score compatible with the level of gait asymmetry identified. Simultaneously, one researcher (A.D.M.) recorded the videos with the AI‐MTS on the opposite side of the vetting line, maintaining a consistent recording height to ensure the horse kept in the centre of the video, without using a tripod for the mobile device. The video recorder operator was positioned approximately 1 metre from the line where the horses were trotting, at a similar distance to the position used by the OEV at the other end. The system was able to analyse the video only if the horse trotted 18–22 strides, and only recordings with at least 20 strides were considered, following the manufacturers' guidelines.

**FIGURE 1:**
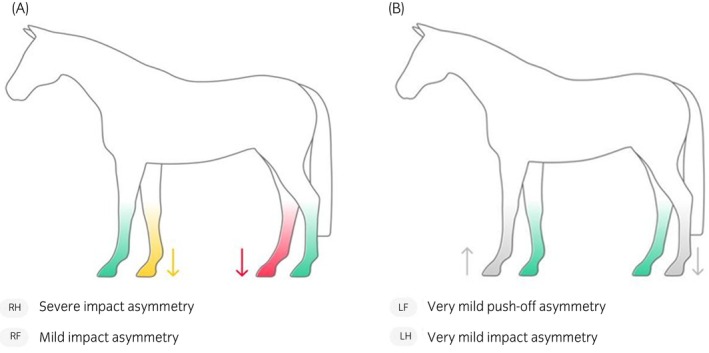
(A) Graphical representation of the asymmetry (no asymmetry = green; mild = yellow; severe = red) by the Sleip AI v1.3.1 (2023). (B) Graphical representation of the asymmetry (no asymmetry = green; very mild = grey) by Sleip AI v1.3.1 (2023).

The uploaded videos were analysed offline with the AI‐MTS neural network system. The system assigned a score and a colour to the asymmetry for each limb: no asymmetry (0–0.2 green), very mild (0.2–0.5 grey), mild (0.5–0.9, yellow), moderate (1.0–1.49, orange), and severe (1.5–2, red) (Figure 1A,B). Horses were categorised according to the described scoring system based on the highest value displayed and the relative colorimetric code, regardless of the affected limb. The AI‐MTS ‘no asymmetry’ and ‘very mild’ categories were classified as ‘no asymmetry’, the ‘mild category’ as ‘mild’, and the ‘moderate’ and ‘severe’ categories as ‘severe’ to allow comparisons between the clinical and the AI‐MTS scoring systems. The OEV and the AI‐MTS operators were blinded to each other's results: they performed their evaluations independently and were not provided with any information regarding the outputs of the counterpart.

### 2.3 Data analysis

The AI‐MTS app and OEV scores were recorded in an electronic spreadsheet. Statistical analysis was performed using commercial software (SPSS 29 for MacOS, SPSS Inc.; JMP Pro, v.17.2, SAS Institute). Normality was tested with the Shapiro–Wilk *W* test, and the data are reported as the means ± standard deviations (SD) or medians (ranges) for normally and nonnormally distributed data, respectively.

Contingency tables and chi‐square tests were applied to explore the associations between the three‐score OEV grading system and the five‐score AI‐MTS grading system for gait evaluation. Whenever significance was detected, each cell's chi‐square (*χ* ^2^) value in the contingency table was examined to identify which specific association contributed the most.^15^ After the described categorisation of the five‐score AI‐MTS into a simplified three‐score system, contingency tables and chi‐square tests were applied again, and the agreement between the OEV and the AI‐MTS gait evaluation methods was assessed using the Fleiss' multi‐rater kappa statistic (*κ*). The strength of agreement was interpreted according to the *kappa coefficient* classified as ‘slight’ (<0.20), ‘fair’ (0.21–0.40), ‘moderate’ (0.41–0.60), ‘substantial’ (0.61–0.80), or ‘almost perfect’ (0.81–1.00).^16^ Power analysis was performed a posteriori with dedicated software (PASS 2024, v. 24.0.2, NCSS LLC), according to the software instructions,^17^ that is, considering the overall agreement identified (*κ* = 0.26) to be different from no agreement (*κ* = 0), with a sample size of 188 videos analysed and with the frequencies reported in Table 1 as the total percentage.

**TABLE 1:** Contingency tables showing the number of evaluations (percentage of the total) in each OEV and AI‐MTS^a^ category (on the left) and modified AI‐MTS category (on the right).

		AI‐MTS categories						Modified AI‐MTS categories				
		No asymmetry	Very mild	Mild	Moderate	Severe	Total		No asymmetry	Mild	Severe	Total
OEV categories	A (*N* = 22)	25 (13%)	86 (45.5%)	43 (23%)	3 (1.5%)	0 (0%)	157 (84%)	A (N = 22)	111 (59%)	43 (23%)	3 (1.5%)	157 (83.5%)
	B (*N* = 157)	1 (0.5%)	8 (4%)	12 (6.5%)	1 (0.5%)	0 (0%)	22 (11.5%)	B (N = 157)	9 (5%)	12 (6.5%)	1 (0.5%)	22 (12%)
	C (*N* = 9)	0 (0%)	0 (0%)	1 (0.5%)	4 (2%)	4 (2%)	9 (4.5%)	C (N = 9)	0 (0%)	1 (0.5%)	8 (4%)	9 (4.5%)
	Total	26 (13.5%)	94 (50.5%)	56 (30%)	8 (4%)	4 (2%)	188	Total	120 (64%)	56 (29.5%)	12 (6.5%)	188

*Note*: Number of horses in each OEV category: (A) soundness (B) mild lameness; (C) severe lameness; in brackets, percentage of total.

Abbreviations: AI‐MTS, Artificial Intelligence Marker‐less motion Tracking System; OEV, Official Endurance Veterinarian.

^a^ Sleip AI v1.3.1 (2023).

To evaluate data variability, for each horse analysed, the mean SD was calculated from the raw data obtained from the software. In particular, the SD of the minimum and maximum vertical positions of the right and left half of a stride for both the forelimbs and hindlimbs were pooled together. Median (range) and the coefficient of variation (CV) of the SD were calculated for the whole sample. The SD was hence categorised as low (≤0.3), medium (≥0.31 to ≤0.6), and high (≥0.61). Both the five‐score and three‐score AI‐MTS simplified categorisation were compared in terms of SD with a Kruskal–Wallis test and an HSD Tukey test. The two AI‐MST categorisations were also evaluated for association to the three SD levels by contingency tables and *χ* ^2^ test.

## 3 RESULTS

This study included 110 horses and 188 videos, leading to a power of 98%. For the category 30 km (debutantes), 30 horses were enrolled for a total of 38 video recordings due to re‐evaluations requested by the OEV; for the 60 km (CEN A) category, 50 horses were enrolled for a total of 82 video recordings; for the 90 km (CEN B) category, 30 horses were enrolled for a total of 68 video recordings. The video recordings did not interfere with the veterinary examination in any case. The OEV assigned 157 (83.5%) A, 22 (12%) B, and 9 (4.5%) C scores.

The overall SD was 0.39 (0.17–0.99) with a CV of 26.8%. According to the SD categorisation, low SD was detected in 29 cases (0.28; 0.17–0.30; 11.5%), medium in 155 cases (0.40; 0.31–0.60), and high in 4 cases (0.83; 0.65–0.99; 23.0%). The median SD did not differ between the original five‐score categories from the AI‐MTS (*p* = 0.15), whereas it significantly differed between the three‐score AI‐MTS simplified categories (*p* = 0.04), with the AI‐MTS ‘mild’ asymmetry category (0.39; 0.29–0.99) displaying a significantly higher SD (*p* = 0.03) compared with the AI‐MTS ‘no asymmetry’ category (0.38; 0.17–0.67). Considering the SD categories, they were not associated with the original five‐score categories from the AI‐MTS (*p* = 0.3), whereas they were significantly associated with the three‐score AI‐MTS simplified categories (*p* = 0.03), with the ‘no asymmetry’ cases overrepresented and the ‘mild’ cases underrepresented in the low SD category.

The original five‐score categorisation system from the AI‐MTS was significantly associated with the OEV evaluation (*p* < 0.0001, *χ* ^2^ = 131; Table 1). In particular, the AI‐MTS moderate and severe categories were highly associated with the C OEV score (*χ* ^2^ = 34 and 76, respectively), and to a lesser extent, the AI‐MTS mild category was associated with the B OEV score (*χ* ^2^ = 5). Moreover, the absence of the AI‐MTS severe category when horses were scored as A and the very mild AI‐MTS category when horses were scored as C by the OEV contributed marginally to the test's significance (*χ* ^2^ = 5 and 4, respectively). When considering the three‐score AI‐MTS simplified categorisation, horses were scored ‘no asymmetry’ in 120 (64%) cases, ‘mild’ in 56 (30%) and ‘severe’ in 12 (6%) cases, and a significant association was identified with the OEV evaluations (*p* < 0.001, *χ* ^2^ = 116; Table 1). In particular, the AI‐MTS ‘severe’ category was highly associated with the OEV C score (*χ* ^2^ = 96) and, to a lesser extent, the ‘mild’ AI‐MTS category with the OEV B score (*χ* ^2^ = 5). Moreover, the absence of the ‘no asymmetry’ AI‐MTS category when horses were scored as C, as well as the small number of the AI‐MTS ‘severe’ category when horses were scored as A by the OEV, contributed marginally to the significance of the test (*χ* ^2^ = 6 and 5, respectively).

The overall agreement between the OEV and AI‐MTS methods was deemed fair. Considering the single categories, substantial agreement was obtained for the ‘severe’ category, whereas only fair agreement was detected for the ‘no asymmetry’ category. The agreement between the two methods was slight and nonsignificant for the ‘mild’ category. The statistical results are summarised in Table 2. An analysis of the limb location data obtained from AI‐MTS revealed that in 22/188 (12%) tests, the AI‐MTS score was 0 for all four limbs. In 35/188 (19%) tests, asymmetry was localised in one or both forelimbs, 45 (24%) in one or both hindlimbs, and 86/188 (46%) in both the forelimbs and the hindlimbs. The specific limb locations of the asymmetry considering the modified AI‐MTS categories for the 166 positive tests are summarised in Table 3.

**TABLE 2:** Overall and within‐category agreement between the clinical and AI‐MTS^a^ gait analysis methods.

	*κ*	SE	95% CI	*p* value
Overall agreement	0.26	0.06	0.14–0.38	<0.001
No asymmetry	0.25	0.07	0.10–0.39	<0.001
Mild	0.13	0.07	−0.02 to 0.27	0.08
Severe	0.75	0.07	0.60–0.89	<0.001

*Note*: Overall agreement on the whole sample, that is, without considering the three categories, between the OEV and software evaluations.

Abbreviations: *κ*, Fleiss kappa coefficient; AI‐MTS, Artificial Intelligence Marker‐less motion Tracking System; CI, confidence interval; OEV, Official Endurance Veterinarian; SE, standard error of *κ*.

^a^ Sleip AI v1.3.1 (2023).

**TABLE 3:** Distribution of the number of tests evaluated according to the anatomic location within the modified AI‐MTS^a^ ‘No asymmetry’, ‘Mild’ and ‘Severe’ categories (percentage of the total).

	No asymmetry (*N* = 98)	Mild (*N* = 56)	Severe (*N* = 12)
Forelimbs	27 (16.5%)	6 (3.5%)	2 (1%)
Hindlimbs	34 (20.5%)	10 (6%)	1 (0.5%)
Forelimbs and hindlimbs	37 (22.5%)	40 (24%)	9 (5.5%)

Abbreviation: AI‐MTS, Artificial Intelligence Marker‐less motion Tracking System.

^a^ Sleip AI v1.3.1 (2023).

The evaluation of the contingency tables between the OEV and the AI‐MTS evaluations considering the limbs affected according to the AI‐MTS output confirmed the significant association between the subjective and the AI‐based systems, especially for the ‘severe’ AI‐MTS and OEV C categories. Cases categorised as ‘severe’ by AI‐MTS and B by the OEV involved the forelimbs in 1 case and both the forelimbs and hindlimbs in 2 cases. Cases categorised as ‘mild’ by AI‐MTS and B by the OEV involved the forelimbs in 5 cases, the hindlimbs in 8 cases, and both forelimbs and hindlimbs in 30 cases. Cases categorised as B by the OEV and ‘no asymmetry’ by AI‐MTS involved the forelimbs in 3 cases, the hindlimbs in 3 cases, and both the forelimbs and hindlimbs in 2 cases.

## 4 DISCUSSION

The AI‐MTS system did not interfere with the regular inspection carried out by the OEV, at least during Italian regional endurance competitions. In contrast to our hypothesis, the overall agreement between the OEV visual lameness examination results and AI‐MTS was fair rather than moderate.

This study also revealed a highly significant association and fair overall agreement between the described gait analysis system and visual lameness evaluation in the sample analysed. This is the first time that the use of this AI‐MTS during endurance competitions has been published.

Elimination rates in endurance competitions can reach 60%,^2^ and lameness is the leading cause of elimination (50%–70%).^18^ An objective and easy‐to‐use system to assess lameness during competitions would be a valuable aid in endurance competitions. In the sample analysed in this study, only 4.5% of the horses were excluded by the OEV because of severe gait asymmetry. Horses that participate in long‐distance competitions, that is, more than 130 km, have the highest risk of elimination for lameness^5^ and the lower exclusion rate due to lameness identified in the present study might be explained by the shorter races selected for sample collection, only up to 90 km.

The AAEP scale (modified to allow half points) and the FEI scale used by the OEV were grouped together by attempting to match the most similar categories possible. The grouping criteria were selected based on the understanding that, according to the AAEP lameness scale, mild lameness (graded 1–2/5) can only be reliably detected under specific conditions, such as during tight circles, rather than in a straight‐line evaluation.^8^ Mild lameness is often not easily observable in straightforward assessments, which is why horses with these grades of lameness were included in the ‘A’ (symmetrical) category. It is important to note that this inclusion does not preclude the possibility that, during a more detailed lameness examination, lameness may become detectable. We adopted the clinical lameness scale, defined by the FEI guidelines, for lameness evaluation in endurance competitions, which ensures consistency and standardisation of assessments across the competition.^1^ Aligning with these official protocols was essential to ensure that the results were consistent with standards for lameness evaluation in this specific context.

The substantial agreement of the AI‐MTS results with the OEV most severe lameness score, supports its strong ability to identify severe gait asymmetries. On the other hand, the OEV considered horses ‘fit‐to‐compete’ when AI‐MTS assigned a ‘moderate’ asymmetry in 2% of the cases. This latter judgement could have led to the exclusion of some participants considering the merging of ‘moderate’ and ‘severe’ asymmetries in this study. Hence, in these instances, the AI‐MTS results might prevent some horses from competing, which in some cases could be seen as a preventive measure.^19^ However, we have no information to indicate that these ‘moderate’ asymmetry cases experienced problems while continuing in the competition and our study was not designed to examine this issue.

Only fair agreement was highlighted in ‘no asymmetry’ cases, and in ‘mild’ cases, slight and non‐significant agreement was detected. This finding agrees with another study where the intra‐rater agreement for live evaluations was fair.^20^ Moreover, the accuracy of visual gait assessment has been deemed unlikely to distinguish between sound and mildly lame horses correctly and to reliably classify horses presenting with gait asymmetry of 20% and below.^8^ However, in horses with a high level of fitness for athletic performance, an inertial measurement unit‐based (IMU) gait analysis system detected asymmetries of the same magnitude as reported in horses with confirmed clinical lameness in a high percentage of horses.^21^ With the development of automated, objective quantification of equine gait able to measure gait asymmetries, clinicians should be cautious in employing the term ‘lameness’ which should be reserved for horses deemed unfit to compete on the basis of a comprehensive assessment that includes but does not rely exclusively on the degree of gait asymmetry.^22^

The AI‐MTS localised asymmetry in the forelimbs in 19% of the 188 recordings, 24% in the hindlimbs, and 46% in both fore‐ and hindlimbs in the present study. It has been reported that clinicians more easily identify lameness affecting the forelimbs than the hindlimbs. Most equine practitioners focus on the vertical movement of the head to identify forelimb asymmetry. In contrast, hindlimb lameness detection is more challenging because of both axial tuber coxae rotation and vertical movement of the pelvis.^8^, ^23^, ^24^ For this reason, correctly identifying the affected limb can be challenging, and misdiagnoses are not uncommon.^6^, ^8^ The individual assessments from the application, which provided data on the location of the lameness, were compared with the veterinarians' scores, which did not take this localisation into account, to see if there was a correlation between the location of the asymmetry and the difficulty in detecting it. Nonetheless, the lack of agreement between the OEV and AI‐MTS does not appear to be related to lameness localisation, as discordance was observed equally in both the forelimbs and hindlimbs.

On the other hand, the discordance rate was higher in the ‘mild’ AI‐MTS category, representing 16% of the whole sample, compared with cases scored as ‘no asymmetry’ by AI‐MTS and as B (mild lameness) by the OEV. This result might explain the only fair agreement in the ‘mild’ AI‐MTS category between the OEV and the AI‐MTS. The gait pattern when multiple limbs are affected by lameness is largely unknown and should be appropriately distinguished from compensatory lameness.^25^, ^26^ Hence, multiple limb involvement, compensatory movements, and a low degree of asymmetry may not have an overall perceivable influence on the horses' gait and may have affected the OEV assessment.

Few studies have explored the application of objective lameness assessment systems during horse competitions. The application of a marker‐based gait analysis system showed varying levels of sensitivity and specificity, from 44.4% to 83.3% and 0% to 66.7%, respectively, compared with OEV during FEI competitions.^20^ A survey questionnaire was submitted to 221 OEVs, revealing that most considered lameness detection and quantification challenging, even for experienced and well‐trained OEVs. Furthermore, most OEVs expressed interest in receiving support from user‐friendly technologies for objective gait evaluation.^27^ Using objective gait analysis during sports competitions may support and improve the subjective assessment of fitness to compete.^10^, ^28^

The variability assessed in this study comes from the same horse repeatedly evaluated at the same time point. Hence, it should not be considered as a measure of data quality, since low‐quality recordings are rejected by the AI‐MTS and not analysed at all. Indeed, a high variation in measurements using a 3D optical motion capture was detected between horses.^29^ Moreover, a high between‐measurements variation was recorded for head movements, probably due to a higher freedom of movement compared with the pelvis.^29^ Hence, the higher variability detected in the present study in the ‘mild’ AI‐MTS category, and without any association to the original five‐score AI‐MTS categorisation, should be rather interpreted as a measure of individual gait differences. Furthermore, this study did not aim to validate the AI‐MTS but focused on evaluating its applicability and comparing its assessments with OEV evaluations during endurance competitions.

Wireless IMUs are considered reliable and precise tools for gait analysis in horses.^30^, ^31^ Previous research compared markerless video analysis with IMU systems for horse gait symmetry assessment, indicating that the AI‐MTS can provide a faster and easier option to set up with meaningful gait data and exhibit asymmetry thresholds similar to those of specialised IMUs.^9^, ^32^ Similarly, the AI‐MTS demonstrated strong concordance with a multicamera motion capture system.^12^ Hence, AI‐MTS should be considered an interesting tool for identifying lameness in horses and is probably the most suitable tool during sports competitions. On the other hand, the use of AI‐MTSs under field conditions has several drawbacks, that is, the requirement for fast internet connections for immediately obtaining results and the consumption of a considerable amount of battery power. In this study, data were initially locally stored on the application, and the analysis was subsequently performed later because of poor internet connectivity in the field. It may be beneficial to consider the use of a portable Wi‐Fi station to obtain the gait analysis promptly. Moreover, using a portable charger connected to the phone would be advisable during data collection.

This study has several limitations. First, the AI‐MTS operator and OEV evaluated the horses from different points, that is, at opposite ends of the trotting line. Even though the AI‐MTS manufacturer recommends examining from the same point of view as the OEV does, the decision to record from a different perspective was made to avoid interfering with the veterinary examination. Moreover, this choice led to a lack of communication between the investigator and the OEV, resulting in the loss of some cases. On the other hand, this ensured that the OEV was masked to the outcome of the video analysis. The AI‐MTS recording device should be located near the OEV if it is to be used as an aid for gait evaluation. Having a single practitioner perform all of the clinical evaluations might be seen as another limitation, but it was made not to introduce a further cause of variability. However, it has been shown that clinicians' years of experience and caseload size do not significantly affect the accuracy of judgements between lame horses and sound horses.^8^ In the future, verifying the agreement of video analysis with the clinical assessment obtained by different OEVs would be interesting. A potential source of error may have been the hand movements of the AI‐MTS operator due to the lack of a tripod to support the phone during video recordings; however, the AI‐MTS developers suggest it only as an option. The recordings were made without a tripod to simulate a typical scenario where a veterinarian might use the system during a live competition, where carrying, setting up, and stabilising a tripod might be less feasible. A constant recording height was maintained relative to the horse movements, minimising the influence of this factor on the results. It would be interesting to compare manually recorded videos with those recorded using a tripod to verify whether there are any differences in the measurements. Finally, when considering the results of the modified three‐score AI‐MTS, merging the ‘moderate’ and ‘severe’ categories should be considered a statistical approach for obtaining 2 three‐score systems for agreement analysis. In this study, the merging was based on the significant association detected for the ‘moderate’ category with the OEV worse lameness score.

## 5 CONCLUSIONS

In conclusion, this markerless system for gait analysis used in the field did not interfere with regular veterinary examinations during endurance competitions. The AI‐MTS displayed fair to substantial agreement with the OEV evaluation. The AI‐MTS can be a helpful and objective aiding tool for OEVs decision‐making when performing visual lameness assessment during endurance competitions, particularly in cases of mild lameness, which is known to be the most challenging to identify. However, the AI‐MTS gait asymmetry results should always be interpreted in light of the clinical examination.

## FUNDING INFORMATION

PhD funds from University of Naples Federico II.

## CONFLICT OF INTEREST STATEMENT

The authors declare no conflicts of interest.

## AUTHOR CONTRIBUTIONS

**Mariaelena de Chiara:** Conceptualization; investigation; writing – original draft; software. **Chiara Montano:** Conceptualization; investigation; writing – original draft; writing – review and editing. **Andrea De Matteis:** Conceptualization; investigation; writing – original draft; software. **Livia Guidi:** Investigation. **Francesco Buono:** Investigation. **Luigi Auletta:** Conceptualization; writing – review and editing; visualization; validation; software; formal analysis; data curation; supervision. **Chiara Del Prete:** Conceptualization; investigation; writing – original draft; writing – review and editing. **Maria Pia Pasolini:** Conceptualization; investigation; writing – review and editing; methodology; formal analysis; supervision.

## DATA INTEGRITY STATEMENT

Mariaelena de Chiara and Luigi Auletta had full access to all the data in the study and take responsibility for the integrity of the data and the accuracy of the data analysis.

## ETHICAL ANIMAL RESEARCH

This study was approved by the Ethical Animal Care and Use Committee of the University of Naples Federico II (protocol number PG/01200450).

## INFORMED CONSENT

Owners gave consent for their animals' inclusion in the study.

## ANTIMICROBIAL STEWARDSHIP POLICY

Not applicable.

## Data Availability

The data that support the findings of this study are available from the corresponding author upon reasonable request: Open sharing exemption granted by the editors.
